# A *copia*-like retrotransposon insertion in the upstream region of the *SHATTERPROOF1* gene, *BnSHP1.A9*, is associated with quantitative variation in pod shattering resistance in oilseed rape

**DOI:** 10.1093/jxb/eraa281

**Published:** 2020-06-11

**Authors:** Jia Liu, Rijin Zhou, Wenxiang Wang, Hui Wang, Yu Qiu, Rosy Raman, Desheng Mei, Harsh Raman, Qiong Hu

**Affiliations:** 1 Oil Crops Research Institute, Chinese Academy of Agricultural Sciences, Wuhan Hubei, P.R. China; 2 NSW Department of Primary Industries, Wagga Wagga Agricultural Institute, PMB, Wagga Wagga, NSW, Australia; 3 The James Hutton Institute, UK

**Keywords:** *BnSHP1.A9*, gene expression, genetic analysis, long terminal repeat retrotransposon, natural variation, oilseed rape, pod shattering, seed

## Abstract

Seed loss resulting from pod shattering is a major constraint in production of oilseed rape (*Brassica napus* L.). However, the molecular mechanisms underlying pod shatter resistance are not well understood. Here, we show that the pod shatter resistance at quantitative trait locus *qSRI.A9.1* is controlled by one of the *B. napus SHATTERPROOF1* homologs, *BnSHP1.A9*, in a doubled haploid population generated from parents designated R1 and R2 as well as in a diverse panel of oilseed rape. The R1 maternal parental line of the doubled haploid population carried the allele for shattering at *qSRI.A9.1*, while the R2 parental line carried the allele for shattering resistance. Quantitative RT-PCR showed that *BnSHP1.A9* was expressed specifically in flower buds, flowers, and developing siliques in R1, while it was not expressed in any tissue of R2. Transgenic plants constitutively expressing either of the *BnSHP1.A9* alleles from the R1 and R2 parental lines showed that both alleles are responsible for pod shattering, via a mechanism that promotes lignification of the *en*b layer. These findings indicated that the allelic differences in the *BnSHP1.A9* gene *per se* are not the causal factor for quantitative variation in shattering resistance at *qSRI.A9.1*. Instead, a highly methylated *copia*-like long terminal repeat retrotransposon insertion (4803 bp) in the promotor region of the R2 allele of *BnSHP1.A9* repressed the expression of *BnSHP1.A9*, and thus contributed to pod shatter resistance. Finally, we showed a *copia*-like retrotransposon-based marker, *BnSHP1.A9*_R2_, can be used for marker-assisted breeding targeting the pod shatter resistance trait in oilseed rape.

## Teaser

Pod shatter resistance of rapeseed is influenced by a *SHP1.A9* allele that contains a *copia-*like retrotransposon insertion in the promoter region, which silences *SHP1.A9* expression epigenetically via DNA methylation.

## Introduction

Oilseed rape (*Brassica napus* L.) is a major source of edible vegetable oil for human consumption and also provides an important energy resource for stock-feed and biodiesel production. Upon maturity, siliques (pods) of oilseed rape open, dehiscing the seed and causing significant yield loss especially when the crop is harvested after it has fully matured (BBCH scale 95). Pod shattering usually accounts for an approximately 10% yield loss on average, but in certain environments it can cause yield loss up to 50% (Kadkol *et al.*, 1984; Wang *et al.*, 2007). In order to reduce such loss, oilseed rape is harvested either manually or mechanically before full maturation of seeds (windrowing). Although this early harvesting strategy can reduce yield losses, the resulting immature seeds often have lower oil content (Østergaard *et al.*, 2006) whilst containing higher levels of chlorophyll. In recent years, farmers have increased the use of combine harvesters to harvest oilseed rape due to the increased costs and shortages of labor. Varieties with improved pod shatter resistance are more amenable to mechanical harvesting and thus would provide a cost-effective and long-term solution for commercial oilseed rape production.

Genetic variation for pod shatter resistance exists in *Brassica napus*, *Brassica rapa*, *Brassica juncea*, and *Brassica carinata* germplasm (Kadkol *et al.*, 1984; Hu *et al.*, 2012; Raman *et al.*, 2014, 2017; Liu *et al.*, 2016), and this can be exploited in breeding programs targeting improved pod shatter resistance in commercial varieties. Genetic studies have revealed that pod shatter resistance is controlled by multiple genes. Several quantitative trait loci (QTLs) associated with this trait have been described in oilseed rape (Hu *et al.*, 2012; Raman *et al.*, 2014; Liu *et al.*, 2016). However, the identification of the genes underlying the QTL for pod shatter resistance in oilseed rape has not been reported yet.

In the model plant Arabidopsis, which belongs to the same family (Brassicaceae) as oilseed rape, a gene network involved in pod development and dehiscence has been elucidated (Ballester and Ferrándiz, 2017). For example, the MADS-box transcription factors *SHATTERPROOF1* (*SHP1*) and *SHATTERPROOF2* (*SHP2*) (Liljegren *et al.*, 2000), a basic helix–loop–helix (bHLH) gene called *INDEHISCENT* (*IND*) (Liljegren *et al.*, 2004), and *ALCATRAZ* (*ALC*) (Rajani and Sundaresan, 2001) have been shown to regulate the differentiation of the dehiscence zone. The activity of valve margin identity genes is repressed by *FRUITFULL* (*FUL*) in the valves (Gu *et al.*, 1998), and *REPLUMLESS* (*RPL*) in the replum (Roeder *et al.*, 2003). Several phytohormones such as auxin, cytokinin, and gibberellin are also essential for pod development and the expression of valve margin identity and *IND* genes (Arnaud *et al.*, 2010; Marsch-Martínez *et al.*, 2012; Larsson *et al.*, 2014; Simonini *et al.*, 2017; Zuñiga-Mayo *et al.*, 2018; Sara *et al.*, 2019). Braatz *et al.* (2018*a*, *b*) have shown that mutations in *IND* and *ALC* homologs are linked with pod shatter resistance in oilseed rape. Ectopic expression of *FUL* gene from Arabidopsis has been shown to result in pod shatter resistance via inhibiting *SHP* expression in *B. juncea* (Østergaard *et al.*, 2006). However, for commercial production of ‘conventional’ oilseed rape, a fine tuning of these genes is required to develop a desirable level of dehiscence.

Comparative mapping studies have shown that the *SHATTERPROOF* paralogs of Arabidopsis (*SHP1* and *SHP2*) are located in the vicinity of the QTL associated with pod shatter resistance on chromosome A9 (designated as *BnSHP1.A9* and *BnSHP2.A9*) in Australian and Chinese oilseed rape populations, including the R1/R2 doubled haploid (DH) population utilized in this study (Raman *et al.*, 2014; Liu *et al.*, 2016). Both *SHP1* and *SHP2* regulate cell differentiation of the valve margin and promote lignification (Liljegren *et al.*, 2000), and due to high levels of sequence identity they are functionally redundant.

In the present study, we cloned the *BnSHP1.A9* gene underlying the QTL *qSRI.A9.1* for pod shatter resistance and characterized its functional role using gene expression analysis, as well as anatomical and transgenic approaches. Our findings suggested that a long terminal repeat (LTR) retrotransposon promoter insertion contributes to pod shatter resistance in oilseed rape by silencing the expression of *BnSHP1.A9* epigenetically via DNA methylation.

## Materials and methods

### Plant materials and evaluation of resistance to pod shattering

For the genetic analysis, we used parental lines R1 (pod shatter resistant), R2 (pod shatter prone), and 96 DH (R1/R2 DH) lines that showed segregation for pod shatter resistance (Liu *et al.*, 2013). In addition, we also used a total of 135 diverse accessions, comprising four winter type, 119 semi-winter type, and 12 spring type (Supplementary Table S1 at *JXB* online) to validate the association between pod shatter resistance index (PSRI) and the *BnSHP1.A9* promoter-specific marker. In 2012 and 2013 winter-cropped environments, the DH lines and accessions of a diverse panel of oilseed rape were planted at Yangluo Research Station, Hubei province, China. All DH lines and diverse accessions were planted in a field following a randomized complete block design with two replications. Each plot (2×1 m) contained three rows and each row had 18 plants. All accessions were evaluated for pod shatter resistance using a random impact test as described previously (Liu *et al.*, 2016).

### Sequence analysis of *BnSHP1.A9*

Total DNA was extracted from the leaves of 4-week-old seedlings using the CTAB method (Stein *et al.*, 2001). The reference sequence of *BnSHP1.A9* (BnaA09g55330D) from the *B. napus* cv. Darmor-*bzh* genome (www.genoscope.cns.fr/brassicanapus) was used as a template to design specific primer pairs (Supplementary Table S2) for cloning the genomic sequence and open reading frame of *BnSHP1.A9*. DNA amplification was performed in a 20 μl reaction comprising 2 μl (10 μM) of forward and reverse primers, 4 μl 5×TransStart^®^FastPfu Fly buffer, 2 μl 2.5 mM dNTPs, 2 μl 5×PCR stimulant, 0.4 μl 50 mM MgSO_4_, 1 U TransStart^®^ FastPfu Fly DNA Polymerase (Beijing TransGen Biotech Co., Ltd, product code: AP231), and 20–30 ng template DNA. After initial denaturation of template DNA at 95 ^o^C for 3 min, PCR was carried out following 34 cycles of 20 s at 94 ^o^C, 30 s at about 5 ^o^C lower than melting temperature of the primer pair used, 1 min kb^−1^ at 72 ^o^C with a final extension of 5 min at 72 ^o^C. The PCR products were separated by electrophoresis in 0.8% low melting agarose gel and slices containing the target bands were purified before cloning into the pEASY-Blunt Zero cloning vector (Beijing TransGen Biotech Co., Ltd, product code: CB501-02). Transformation was carried out using *Trans*1-T1 competent *Escherichia coli* cells with the heat shock method (Beijing TransGen Biotech Co., Ltd, product code: CB501, CT101). Four to six positive clones were randomly selected and sequenced with M13 sequencing primers at Shanghai Sangon Biotech Co., Ltd (http://www.sangon.com/). Cloned DNA sequences were analysed by MultAlin online software (Corpet, 1988). BLASTn was used to determine the genomic locations and sequence similarities of *BnSHP1.A9* clones by comparison with the Darmor-*bzh* reference sequence database (http://www.genoscope.cns.fr/brassicanapus/). The upstream sequence of BnSHP1.A9 was cloned using two specific primer pairs (listed in Supplementary Table S2).

### Development of *BnSHP1.A9*-specific marker

MEGA7 (Kumar *et al.*, 2016) was used to align the genomic sequences of *BnSHP1.A9* from R1 and R2 to the reference genomic and coding sequences (BnaA09g55330D) of Darmor-*bzh*. Based on the sequence differences we developed a co-dominant marker (IF4) that identified an insertion/deletion (InDel) difference in the first intron of *BnSHP1.A9* between the parental lines of the R1/R2 population. This marker was used for subsequent genetic mapping and the allelic diversity analysis (Supplementary Table S2). PCR was performed in a volume of 10 μl, including 5 μl 2× Taq MasterMix, 1 μl (10 μM) of each primer, 2 μl ddH_2_O, and 1 μl genomic DNA (20–30 ng). The PCR condition used was as follows: 3 min at 94 ^o^C, 35 cycles of 30 s at 94 ^o^C, 30 s at 57 ^o^C, 30 s at 72 ^o^C, with a final extension of 5 min at 72 ^o^C. The PCR products were examined on either 3% agarose gel by electrophoresis at 130 V for 1 h or using capillary electrophoresis on an automated CEQ2000 system (Beckman-Coulter) as described previously (Raman *et al.*, 2005). The gels were stained with SYBR Green (TransGen Biotech Co., Beijing) and visualized under UV light. The PCR yielded a 267-bp product from R1 (pod shatter resistance line) and a 290-bp product from R2 (pod shatter prone line).

### Quantitative trait locus mapping

The 96 DH lines derived from the R1/R2 cross were genotyped with the InDel marker IF4 and other new InDel and simple sequence repeat (SSR) markers developed based on sequence variation between the two parental lines in the vicinity of the *qSRI.A9* region (Supplementary Table S3). The InDel and SSR markers were integrated with the Illumina Brassica 60K Infinium ® SNP array and pod shatter resistance data, generated in our previous study (Liu *et al.*, 2016). To determine putative QTLs for pod shatter resistance, we performed composite interval mapping using WinQTL Cartographer 2.50 (Zeng, 1994; Wang *et al.*, 2006). The genome scan was performed at every 2 cM to estimate the likelihood of a QTL and its corresponding phenotypic effect (*R*^2^). The empirical threshold was computed using 1000 permutations (overall error level 5%) as described in Churchill and Doerge (1994).

### Analysis of *BnSHP1.A9* transcript levels

To detect the spatial and temporal expression patterns of *BnSHP1.A9*, tissues from root, stem, and young leaf were collected from 4-week-old plants. Samples from bud, fully open flower, and developing pods were also collected at 10, 20, 30, and 40 d after flowering (DAF) from R1 and R2 parental and DH lines (DH56 and DH82). For each tissue and development stage, three biological replicates were collected with the resulting plant material immediately snap frozen in liquid nitrogen. Total RNA was extracted using the RNAprep Pure Plant Kit (Tiangen Biotech Beijing Co., Ltd, product code: DP432). DNAse I-treated RNA was reverse transcribed using the cDNA synthesizing kit following the manufacturer’s instructions (Tiangen Biotech Beijing Co., Ltd, product code: KR106-02). To eliminate interference from other paralogs in the semi-quantitative reverse transcription PCR (RT-PCR), an allele-specific primer pair *BnSHP1.A9*-RT (F/R) was designed (Supplementary Table S2) with specificity confirmed by sequencing the PCR product. The *actin* gene was used as an internal control for semi-quantification of relative expression levels.

### Generation and analysis of transgenic lines overexpressing *BnSHP1.A9*

To examine the function of different *BnSHP1.A9* alleles from R1 and R2 parental lines, two overexpression vectors driven by the CAMV35S promoter were constructed. 35S::*BnSHP1.A9*_R1_ and 35S::*BnSHP1.A9*_R2_ were generated by cloning the *BnSHP1.A9* coding sequence from R1 and R2 into the pCAMBIA-1301 vector using *Nco*I–*Bst*EII cloning sites. *BnSHP1.A9* cDNA was amplified from developing pods using the primer-pair BnSHP1.A9orf (Supplementary Table S2). The two overexpression vectors were then transformed into *Agrobacterium tumefaciens* (GV3101 strain) individually and then used for transformation of the oilseed rape line R1 (resistant to pod shatter) (Hood *et al.*, 1993). Transgenic T_0_ and T_1_ plants were confirmed by PCR detection of the hygromycin resistance gene with primer pair *HptII* F/R (Supplementary Table S4). The expression level of *BnSHP1.A9* in transgenic plants was evaluated by RT-PCR. As the CAMV35S promoter drives constitutive expression of downstream target genes in all tissues of transgenic plant, for the convenience of RNA extraction and early detection, leaf tissue was used for determining the expression level of *BnSHP1.A9* in transgenic plants. Transgenic lines of the T_1_ generation derived from both constructs were evaluated for pod shatter resistance using the random impact test as described previously (Liu *et al.*, 2016).

### DNA methylation analysis

McrBC endonuclease (NEB, M0272S) was used to investigate the DNA methylation status of the *BnSHP1.A9* promoter region and genic region. Genomic DNA (0.5 µg) was extracted from 20 DAF siliques of R1 and R2 and digested with 5 U McrBC overnight at 37 ^o^C. Methylated plasmid DNA was used as positive control in this experiment. The McrBC-digested DNA was then used as a template to amplify the *BnSHP1.A9* promoter and genic region with allele-specific primer pairs (Supplementary Table S5). The Chop PCR products were then checked by agarose gel electrophoresis and stained with SYBR Green and visualized under UV light. Detailed analysis of promoter DNA methylation was then performed with bisulfite sequencing method (Gruntman *et al.*, 2008). Genomic DNA of R1 and R2 from 20-DAF siliques was bisulfite treated using the EpiTect Bisulfite Kit (Qiagen 59104) following the manufacturer’s instructions. Bisulfite sequencing primers were designed with the MethPrimer online tool (http://www.urogene.org/cgi-bin/methprimer/methprimer.cgi; Li and Dahiya, 2002) and the primer designing tool at the NCBI website. The bisulfite-treated DNA was used as a template to amplify the target fragments of R1/R2 promoter with specific bisulfite primers (Supplementary Table S6) and the resulting PCR fragments were cloned into pTOPO-T simple vector (Aidlab CV1501). At least 24 positive clones were sequenced from each fragment. Vector sequences together with primer sequences of the sequencing results were first trimmed and the remaining sequences were then blasted against the *B. napus* reference genome database (http://www.genoscope.cns.fr/brassicanapus/) to confirm the specificity. The methylation status of the specifically amplified fragments was then analysed by the Kismeth online tool (http://katahdin.mssm.edu/kismeth/revpage.pl). The data for methylated cytosines (CG, CHG and CHH) between the two parental lines were collected and compared by Student’s *t*-test.

## Results

### Sequence variation of BnSHP1.A9 underlying pod shatter resistance

**Fig. 1.**
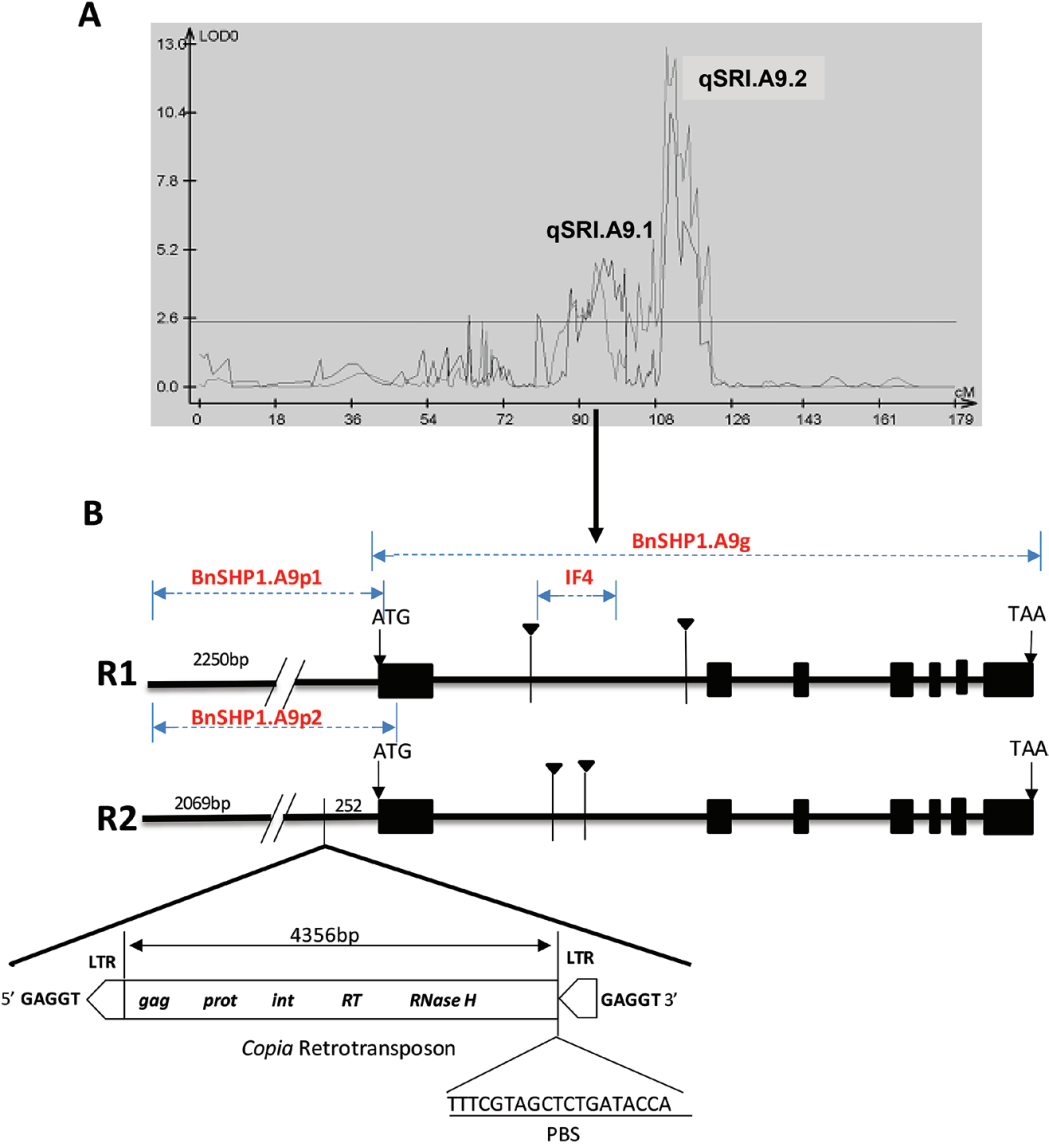
Genetic mapping of the QTL *qSRI.A9.1* and underlying the candidate *SHATTERPROOF1* paralog of Arabidopsis in *B. napus*, *BnSHP1.A9*. (A) Schematic diagram showing the presence of two QTLs for pod shatter resistance, *qSRI.A9.1* and *qSRI.A9.2*, in a doubled haploid (DH) mapping population from R1/R2 on A9 linkage group. The *x*-axis represents the genetic positions of molecular markers on A9, and the *y*-axis represents to the LOD value for association between molecular markers and pod shatter resistance index, measured using random impact test (Liu *et al.* 2016). (B) Schematic diagram of *BnSHP1.A9* alleles of the parental lines of DH from R1/R2, used for genetic analysis of pod shattering resistance. The exon size is indicated in the black boxes and triangles represent InDels. The 4803-bp *copia*-like retrotransposon with LTR inserts in the *BnSHP1.A9* promoter region of R2. QTL was mapped using *BnSHP1.A9* gene-specific InDEL marker IF4, which targets intron 1. *BnSHP1.A9g*-specific primer was used to amplify *BnSHP1.A9* gene spanning exon 1 to exon 7. BnSHP1.A9p1/p2-specific primers were used to amplify the promoter and part of exon 1 sequences to reveal variation in LTR between the parental lines of DH population (R1 and R2).

Pod shatter tolerance is a complex quantitative trait and is affected by genotype by environment (such as temperature and plant maturity) interaction. In this study, we focused on pod shatter resistance at the full maturity stage when more than 90% of the seed turned black. We isolated the genomic sequences of the *BnSHP1.A9* gene from R1 and R2 parental lines of the R1/R2 DH mapping population. The size of *BnSHP1.A9* genomic sequences varied from 2737 bp in R1 to 2757 bp in R2, with seven exons and six introns (Fig. 1B). Sequence alignments revealed polymorphisms in the form of SNPs and InDels between the two parental lines. There were nine SNPs in the exon sequences, including two non-synonymous SNPs in exon 1 at no. 25 (T/G) and no. 203 (G/T), leading to tyrosine (Y) to aspartic acid (D) and arginine (R) to leucine (L) amino acid variation, respectively. The point mutation in exon 3 (no. 1751, T/C) causes a Y to H (histidine) amino acid alteration, while A/T mutation at no. 2480 in exon 6 leads to a D to valine (V) amino acid change (Supplementary Table S7). However, the most abundant sequence divergence was found in the first intron including 21 SNPs and 10 InDels between R1 and R2, which enabled us to develop an allele-specific marker for *BnSHP1.A9*.

In order to determine whether the polymorphism in *BnSHP1.A9* is associated with pod shatter resistance in a R1/R2 DH population, we developed a primer pair (IF4) for specific amplification of *BnSHP1.A9* alleles (Supplementary Table S2). IF4 amplified a 267-bp fragment from R1 and a 290-bp fragment from R2. Genotypic analysis of all the 96 DH lines showed segregation for a single locus, with 35 lines containing the R1 allele and 55 lines containing the R2 allele (χ ^2^=4.44, *P*=0.035) (Supplementary Table S8). The marker data were integrated with previously obtained SNP data from the R1/R2 DH population (Liu *et al.*, 2016). QTL analysis identified two genomic regions, *qSRI.A9.1* and *qSRI.A9.2*, for pod shattering resistance on chromosome A9. *qSRI.A9.1* and *qSRI.A9.2* QTLs explained 11.26–12.07% and 27.58–38.11% of the phenotypic variation, respectively (Fig. 1A; Table 1). At *qSRI.A9.1*, the R2 allele had a positive effect on pod shatter resistance, whereas the R1 allele had a negative effect. In contrast, at *qSRI.A9.2*, the R1 allele had a positive effect on pod shatter resistance, whereas the R2 allele had a negative effect. Linkage analysis revealed the order of markers to be ni113, *BnSHP1.A9*, BrEMS4 (Supplementary Table S3); *BnSHP1.A9* was mapped within the *qSRI.A9.1* genomic region for pod shatter resistance (Table 1; Fig. 1A).

**Table 1.** QTLs associated with pod shatter resistance in a doubled haploid (DH) population from R1 (pod shatter resistant)/R2 (pod shatter susceptible)

QTL	Year	Confidence interval (cM)	LOD score	*R* ^2^ (%)	Additive effect (parental allele)
*qSRI.A9.1*	2013	86.5–93.2	4.91	11.26	−0.07 (R2)
	2014	88.1–96.6	4.76	12.07	−0.15 (R2)
*qSRI.A9.2*	2013	109.6–113.5	13.43	38.11	0.12 (R1)
	2014	109.0–112.1	10.32	27.58	0.23 (R1)

DH lines were grown across two field environments in 2013 and 2014 and evaluated for pod shatter resistance using random impact test (Liu *et al.*, 2016). *R*^2^ refers to phenotypic variance explained. LOD score refers to statistical significance for association between molecular markers and phenotype (pod shatter resistance index).

### Expression of BnSHP1.A9 is repressed in R2

**Fig. 2.**
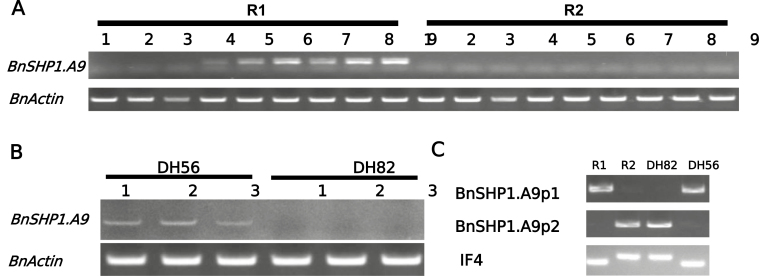
*BnSHP1.A9* expression levels correlates with LTR retrotransposon insertion in the parental lines (R1 and R2) and selected two DH lines derived from R1/R2. (A) *BnSHP1.A9* expression analysis of different tissues and siliques, taken at different developmental stages of R1 and R2. *BnActin* gene was used as an internal control for relative expression analysis. 1: root; 2: stem; 3: leaf; 4: bud; 5: flower; 6: 10 d silique; 7: 20 d silique; 8: 30 d silique; 9: 40 d silique. (B) *BnSHP1.A9* expression status in DH56 and DH82 lines. 1: 10 d silique; 2: 20 d silique; 3: 20 d silique. (C) Genotypes of the R1, R2, DH56, and DH82 lines with *BnSHP1.A9* promoter-specific primers (BnSHP1.A9p1/p2) and InDel primer pair (IF4).

To test the dynamic expression of *BnSHP1.A9*, we investigated its expression pattern in root, stem, leaf, bud, flower, and developing siliques in R1 and R2, the parental lines of the mapping population. Since there is another homologous copy of *SHP1* on chromosome C08 (BnaC08g29520D), to eliminate potential non-specific amplification, we designed a *BnSHP1.A9-*specific primer pair for RT-PCR based on SNPs between coding sequences of these two homologs. The result showed that *BnSHP1.A9* was expressed exclusively in bud, flower, and developing siliques in R1. In contrast, almost no expression in either vegetative organs or reproductive organs could be detected in R2 (Fig. 2A). Differential *BnSHP1.A9* expression hints that the level of pod shatter resistance may be enhanced by manipulating *SHP1* expression in oilseed rape.

### Overexpression of BnSHP1.A9 alleles promoted pod shattering in the pod shatter-resistant R1 line

**Fig. 3.**
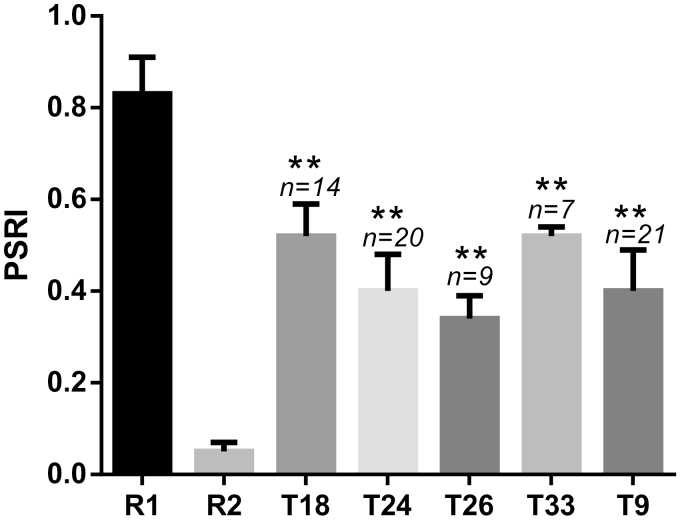
Genetic variation in pod shatter resistance index (PSRI) of different transgenic (T1) plants (T18, T24, T26, T33, and T9) and two parental lines (R1 and R2) of a doubled haploid mapping population from R1/R2. PSRI was assessed with random impact test, using 20 pods from each line in three replicates. Standard deviation of each line is also shown. Statistical significance was determined by analysis of variance; **P<0.01.

Sequence analysis of *BnSHP1.A9* revealed four non-synonymous SNPs in the coding sequence of the two *BnSHP1.A9* parental alleles, three of these located in the predicted conserved MADS-box and K-box domains. To verify the function of *BnSHP1.A9*, we overexpressed the coding sequence of *BnSHP1.A9*_R1_ or *BnSHP1.A9*_R2_ alleles in R1 (pod shatter resistant line). A total of 71 transgenic plants were generated and subsequently assessed for the PSRI by random impact test. The PSRI of the transgenic T1 plants varied from 0.35 to 0.53, in comparison with the ‘wild-type’ (untransformed) R1 plants, averaged 0.83 (*n*=8) (Fig. 3). However, no statistically significant differences were found for PSRI between the transgenic *BnSHP1.A9*_R1_ and *BnSHP1.A9*_R2_ overexpression (OE) lines. For example, the four OE lines T18, T24, T26, and T33 of *BnSHP1.A9*_R1_ had similar PSRI to that of the T9 line of *BnSHP1.A9*_R2_ (Fig. 3; Supplementary Table S9).

**Fig. 4.**
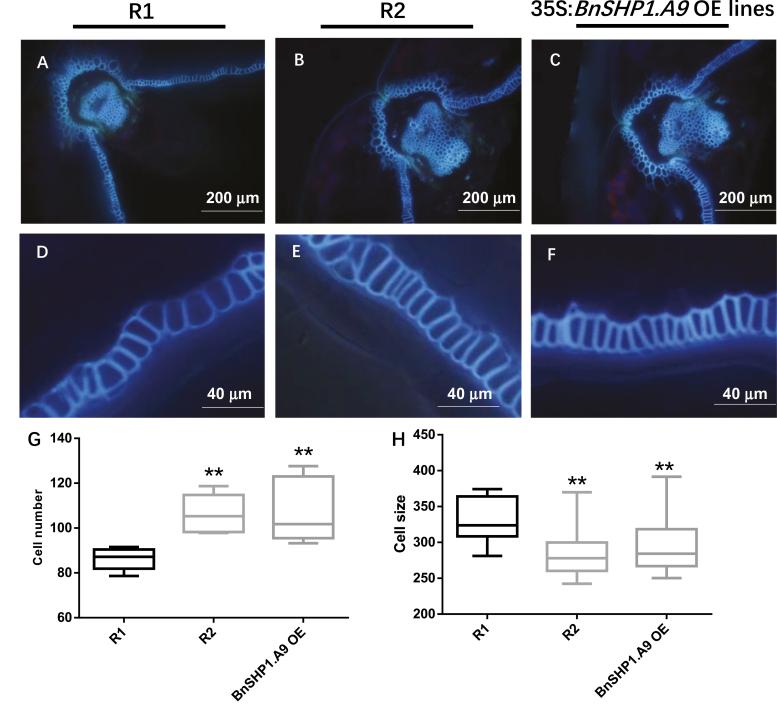
Cross-sections of siliques, collected after 30 d of pollination from R1, R2, and 35S:*BnSHP1*.*A9* transgenic oilseed rape lines. Sections were visualized under fluorescence microscopy. (A–F) Lignified cells in the ‘*en*b’ layer of R1 (A, D), R2 (B, E) 35S:*BnSHP1.A9* (C, F) transgenic oilseed rape siliques. Scale bars, 200 and 40 μm. (G, H) Variation in cell number (G) and cell size (H) between R1 and R2/*BnSHP1.A9* OE lines. Data obtained from 10 individuals of each test line. Error bars, SD; statistical significance was determined by a two-sided *t*-test; **P<0.01.

Expression of *BnSHP1.A9* in developing pods in 15 selected transgenic T1 plants (three from each T1 line) was examined using semi-quantitative RT-PCR. All the tested T1 plants exhibited an elevated level of *BnSHP1.A9* expression (Supplementary Fig. S1). These results indicate that both alleles of the *BnSHP1.A9* gene (R1 and R2) can promote pod shattering in oilseed rape. The overexpression of *BnSHP1.A9* in transgenic plants did not alter flowering time and duration of flowering (Supplementary Fig. S2), suggesting that the *BnSHP1.A9* gene does not have any pleiotropic effects on flowering time. To explore whether overexpression of *BnSHP1.A9* causes dehiscence zone differentiation in siliques, we analysed the anatomical structure of pods from *BnSHP1.A9*_R1_ and *BnSHP1.A9*_R2_ OE lines, as described in Raman *et al.* (2017). A more compact arrangement of the ‘*en*b’ layer at the valve margin was observed in cross sections of the pods derived from *BnSHP1.A9*_R1_ and *BnSHP1.A9*_R2_ OE T1 lines than in the R1 genotype (Fig. 4). Our results indicate that the *BnSHP1.A9* gene promotes pod shattering through an increase in the number and compression of the cell size of lignified cells in the ‘*en*b’ layer, thus enhancing tension caused by differential contraction of the fruit wall tissue in oilseed rape.

### A long terminal repeat retrotransposon is inserted in BnSHP1.A9 promoter of R2

To gain an understanding of the promoter region of the two *BnSHP1.A9* alleles, we sequenced and analysed the upstream region of *BnSHP1.A9* from both parental lines of the mapping population. We identified a 4803 bp LTR/*copia-*like retrotransposon insertion at 252 bp upstream the start codon of *BnSHP1.A9* in R2 in the opposite orientation (Fig. 1B). This LTR retrotransposon contains the typical retrotransposon structure, including a predicted 4356 bp single open reading frame with conserved *gag*, *prot*, *int*, *RT*, and *RNaseH* domains, and two identical 169 bp 5′ and 3′ LTRs flanked by a 5 bp direct repeat sequence (5′-GAGGT-3′) (Cao *et al.*, 2015). Sequence alignment of this LTR retrotransposon against the NCBI database revealed 100% sequence identity with a *B. rapa* A9 scaffold (LR031568.1), which suggested that this LTR retrotransposon insertion may have originated from *B. rapa* in the R2 paternal line of the R1/R2 DH mapping population.

As the LTR element is inserted upstream of the start codon of the *BnSHP1.A9*_R2_ allele, it may play a role in the repression of the *BnSHP1.A9* gene. To further test this hypothesis, we investigated the 96 R1/R2 DH lines using promoter-specific primers of *BnSHP1.A9* and selected two DH lines, DH56 (LTR^−^, PSRI=0) and DH82 (LTR^+^, PSRI=0.85), which had the same genotype as *BnSHP1.A9*_R1_ and *BnSHP1.A9*_R2_, respectively. We then collected 10, 20, and 30 DAF siliques and analysed *BnSHP1.A9* expression level in siliques of DH56 and DH82 lines by RT-PCR. A weak band was visible in the siliques of all three developmental stages of DH56, which does not contain the LTR insertion (LTR^−^), while there was no amplification in any of the three silique samples of DH82 containing the LTR insertion (LTR^+^) (Fig. 2B). Based on the expression patterns of *BnSHP1.A9* in the two parental lines, our results suggest that the LTR insertion upstream of *BnSHP1.A9* is responsible for repression of *BnSHP1.A9* transcript (resistance to pod shatter) in the DH82 line.

### DNA methylation of long terminal repeat retroelement insertion is responsible for the repression of BnSHP1.A9

**Fig. 5.**
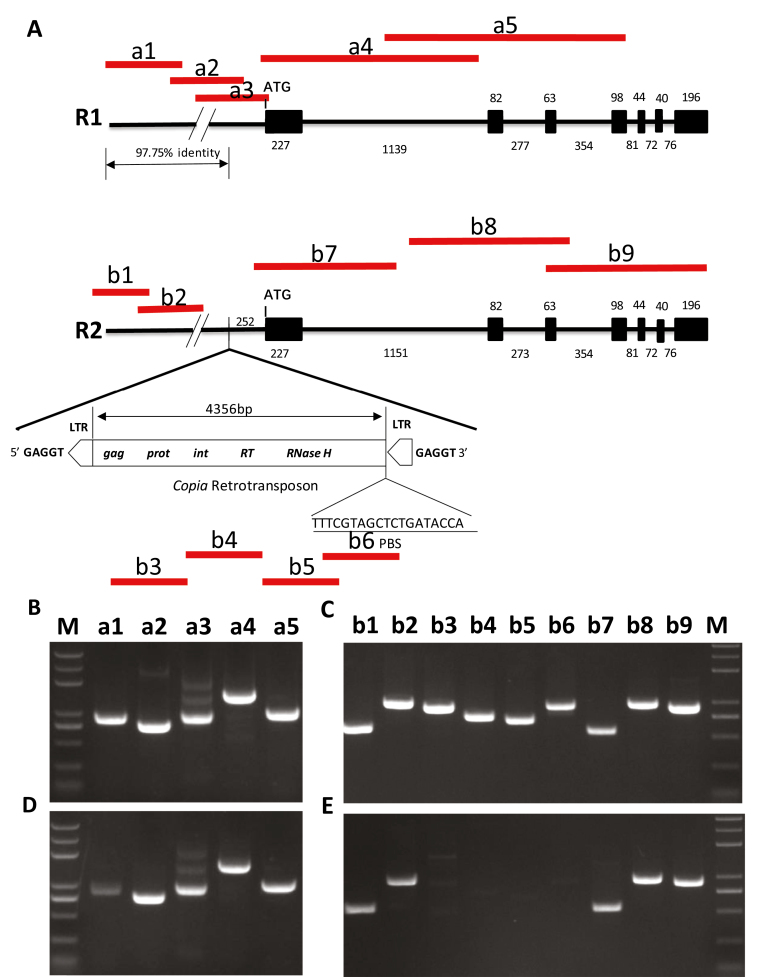
Cross validation for DNA methylation status of *BnSHP1.A9* alleles. Siliques were collected from the R1 and R2 parental lines of a doubled haploid mapping population used for genetic analysis. (A) Diagram of amplified fragments (shown by the lines) in *BnSHP1.A9* alleles from the parental lines R1 and R2. Fragments varying in size are shown in R1 line (a1: −2412/−1542; a2: −1616/−882; a3: −902/−30; a4: −49/+1413; a5: +1034/+2007) and in R2 line (b1: −7412/−6823; b2: −6753/−5765; b3: −3191/−2285; b4: −2538/−1788; b5: −1707/−1027; b6: −1035/−102; b7: −49/+436; b8: +1035/+2012; b9: +1883/+2793). (B, C) Chop-PCR analysis of *BnSHP1.A9* alleles from R1 (B) and R2 (C). Amplicon was not digested with McrBC (as control). (D, E) Chop-PCR analysis of *BnSHP1.A9* alleles from gDNA McrBC digested from R1 (D) and R2 (E). Siliques were collected 20 DAF and used for chop-PCR analyses (with McrBC digestion). M: Trans2K PlusII DNA Marker.

**Fig. 6.**
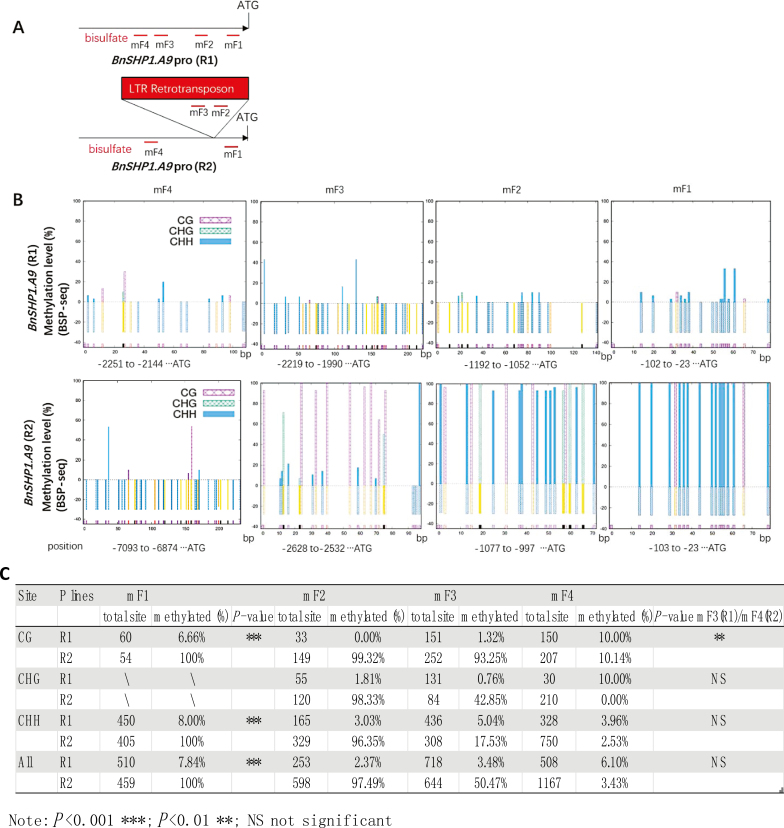
Methylation status of upstream promoter regions of the *BnSHP1.A9* alleles of R1 and R2, revealed by bisulfite sequencing (BSP) analyses. (A) Methylation marks in the upper 5′ UTR region of *BnSHP1.A9* (black line) and 4803 bp *copia* LTR-retrotransposon insertion (red box). Red lines indicate the positions targeted for the bisulfite sequencing (BSP) analyses. (B) Methylation sites of cytosine residues are shown in different colors: CG (purple), CHG (green), and CHH (blue). (C) Methylation data of parental lines of a doubled haploid population (R1 and R2) showing details of cytosine residues in CG, CHG, and CHH sites from the four BSP regions.

To understand the basis of LTR insertion-mediated repression of *BnSHP1.A9*, we performed site-specific chop-PCR and bisulfite sequencing of genomic DNA extracted from 20 DAF siliques of R1 and R2 to analyse the DNA methylation status of the upstream and genomic regions of *BnSHP1.A9*. Chop-PCR amplified no bands from the LTR insertion fragments (b3, b4, b5, and b6) of R2 (Fig. 5C, E) because of hypermethylation of the sequences, whereas the same bands were amplified from other fragments of R2 similar to R1 (Fig. 5B, D). These observations hinted that DNA methylation mainly occurred in the LTR insertion. The methylation of cytosine residues of the LTR inner region were maintained at a longer distance from the central insertion region. The bisulfite sequencing results also showed that the LTR retrotransposon and the transcription start region of *BnSHP1.A9* in R2 was hypermethylated, while the promoter region of *BnSHP1.A9* in R1 was hypomethylated (Fig. 6B). The methylation levels of the mF4 region in R2 located at about 1.8 kb upstream of the LTR element and the corresponding region of mF3 in R1 are much lower than those of the downstream regions close to the start codon (Fig. 6B, C). In the mF1 region, we found that the 100% cytosine (C) residues in all sites of CG and CHH (H represents any residues other than G) were methylated in R2, whereas only 7.84% of the cytosine residues of the corresponding sites were methylated in R1. The mF2, mF3, and mF4 regions in R1 were hypomethylated from 2.37 to 6.10%. We investigated the methylation level of mF2 and mF3 of the LTR region in R2. In the mF2 region, 97.49% of cytosine residues were hypermethylated, while only 50.47% of cytosine residues were methylated in the mF3 region. Among these cytosine residues containing sites in the mF3 region, cytosine residues of CG were still hypermethylated, but the methylation of cytosine residues of CHG and CHH were significantly decreased to 43.85% and 17.53%, respectively. For mF4, in the upstream region about 2 kb away from the LTR and 7 kb away from the transcription start codon of *BnSHP1.A9*, the methylation level was significantly decreased to a normal level of about 6.10% in both lines. Thus, methylation of the LTR retrotransposon in the vicinity of the *BnSHP1.A9* promoter region in R2 may directly affect in the down-regulation of *BnSHP1.A9* expression.

### The long terminal repeat insertion correlates with pod shatter resistance in diverse oilseed rape germplasm

**Fig. 7.**
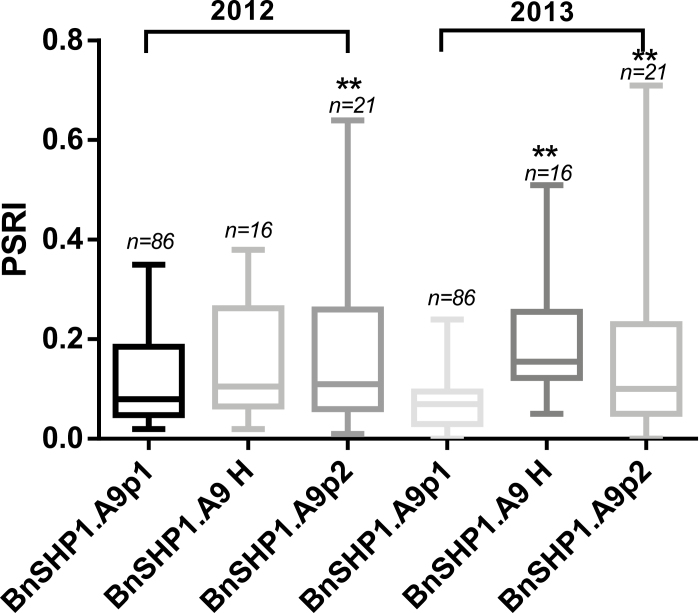
Frequency of *BnSHP1.A9* alleles associated with pod shatter resistance index (PSRI) in oilseed rape. The PSRI was determined among 135 oilseed rape accessions, grown under two environments (2012 and 2013). PSRI was assessed using random impact test, as described previously (Liu *et al.* 2016). BnSHP1.A9p1, the alleles (86 accessions) of *BnSHP9.A1* (R1) promoter in 135 rapeseed accessions; BnSHP1.A9p2, the LTR insertion alleles (21 accessions); BnSHP1.A9 H, the heterozygote of the two alleles (16 accessions). In box plots, central values are medians, lines indicate variability outside the upper and lower quartiles. Statistical significance was determined by analysis of variance; **P<0.01.

To verify the linkage between this LTR retrotransposon insertion and pod shatter resistance, we tested 135 diverse accessions with an allele-specific diagnostic marker for LTR (BnSHP1.A9p1/p2, Supplementary Table S1). The homozygous *BnSHP1.A9* (R1) allele (R1 specific, LTR^−^) and homozygous *BnSHP1.A9* (R2) allele (R2 specific, LTR^+^) were detected in 63.7% (86) and 15.6% (21) of the accessions, respectively, and the heterozygous *BnSHP1.A9* (H) allele was found in 11.9% (16) of the accessions (Supplementary Table S1). A significant association between the *BnSHP1.A9* (R2, LTR^+^) promoter allele and PSRI was observed among the tested oilseed rape accessions (Fig. 7). The average PSRI of the lines with *BnSHP1.A9* (R2, LTR^+^) genotype was significantly higher than that of lines with *BnSHP1.A9* (R1, LTR^−^), indicating that the *BnSHP1.A9* (R2, LTR^+^) allele could increase the PSRI compared with *BnSHP1.A9* (R1, LTR^−^) allele.

## Discussion

### SHATTERPROOF1 gene, BnSHP1.A9, underlies pod shatter resistance

In this study, we identified two QTLs, *qSRI.A9.1* and *qSRI.A9.2*, with large allelic effects on chromosome A9 in a DH population from R1/R2 (Fig. 1). The QTLs, accounting for a large proportion of the phenotypic variation of pod shatter resistance, have also been identified previously in oilseed rape populations (Hu *et al.*, 2012; Raman *et al.*, 2014; Liu *et al.*, 2016). Since a *SHP1* paralog of Arabidopsis is located in the vicinity of the significant SNP markers associated with pod shatter resistance on chromosome A9 (Raman *et al.*, 2014; Liu *et al.*, 2016; this study), we were interested in determining whether *SHP1* contributes to the genetic variation for pod shatter resistance in diverse oilseed rape germplasm. Through genetic analyses (using a DH population from R1/R2 and a set of 135 diverse lines of oilseed rape breeding germplasm) and functional characterization via comparative expression analysis and transgenic approaches, we showed that the *SHP1* paralog *BnSHP1.A9* underlies pod shatter resistance at *qSRI.A9.1* in oilseed rape.

Our results revealed that *BnSHP1.A9* is the functional gene regulating pod shatter resistance in oilseed rape. By overexpressing *BnSHP1.A9* cDNA from both R1 and R2 alleles, an average 50% decrease in PSRI was observed in T1 lines, thus confirming the function of *BnSHP1.A9* in pod shatter regulation in oilseed rape (Fig. 3). The observations of the functional analysis are in accordance with the expression pattern of *BnSHP1.A9* in the R1 and R2 parental lines. In R2, which has a positive effect on the PSRI at this locus, the expression of *BnSHP1.A9* was repressed, indicating that the down-regulation of the target gene enhances the PSRI. Overexpression of *BnSHP1.A9* only partly decreased the pod shatter resistance of R1. This can be attributed to other loci known to have a positive effect in R1 for pod shatter resistance, such as *qSRI.A9.2* as well as other *SHP1* or *SHP2* homologs that are known to act redundantly and control dehiscence zone differentiation (Liljegren *et al.*, 2000). Although an expression difference of *BnSHP1.A9* exists in lines with contrasting genetic effects of this locus, the allelic variation of *BnSHP1.A9 per se* is not the causal factor for the phenotypic variation of pod shatter resistance, as overexpression of both alleles in the *BnSHP1.A9* gene facilitated pod shattering in oilseed rape.

### LTR retrotransposon insertion in the upstream region regulates BnSHP1.A9 epigenetically

Comparative and association analyses revealed that a LTR retrotransposon insertion was significantly associated with pod shatter resistance among 135 collected accessions and in the R1/R2 DH population. The promoter region of the *BnSHP1.A9*_R2_ allele, including the LTR retrotransposon insertion, was found to be highly methylated (Fig. 6), which is responsible for the repression of *BnSHP1.A9* expression and the positive effect on pod the shatter resistance phenotype. Transposable elements (TEs) are well known to play positive roles in generating genomic novelty and diversity in plants (Song and Cao, 2017). TEs are frequently found in *B. napus* genomes (Chalhoub *et al.*, 2014; Sun *et al.*, 2017) and have been implicated in DNA methylation and H3K9me2 modification (Eichten *et al.*, 2012; Gent *et al.*, 2013), altering gene expression both genetically and epigenetically (Cui and Cao, 2014). In fact, several oilseed rape genes related to morphological or physiological traits have evolved from TE insertions (Hou *et al.*, 2012; Zhang *et al.*, 2015; Gao *et al.*, 2016; Shi *et al.*, 2019). The current study implies that DNA methylation of the *copia*-like retrotransposon insertion spreads to the *BnSHP1.A9 cis*-regulatory region. This epigenetic modification may change the accessibility of RNA polymerase II and transcription factors to the *BnSHP1.A9* promoter, ultimately altering transcription patterns (Zhao *et al.*, 2013).

*Brassica napus* is an allotetraploid originated from natural hybridization of *B. rapa* and *B. oleracea*. The LTR insertion in the *BnSHP1.A9* promoter region showed 100% sequence identity with *B. rapa*. This suggests that the insertion of the LTR retrotransposon may have occurred before the generation of *B. napus* as a species. However, only 15.6% of the natural *B. napus* population was found to contain the LTR insertion, which indicates that the LTR insertion might have either been lost in the process of domestication or breeding of oilseed rape. As pod shattering is beneficial to seed release, the loss of the LTR insertion is evolutionarily advantageous. This is further consistent with the theory that pod shatter resistance was an adverse selection during natural evolution or domestication (Raman *et al.*, 2014). We thus propose a simple model to explain *BnSHP1.A9*-dependent pod shatter resistance in oilseed rape. The non-LTR *BnSHP1.A9* (in R1, LTR^−^) is in a transcriptionally active state. DNA methylation of the LTR insertion in the *BnSHP1.A9* promoter (in R2, LTR^+^) spreads to the transcription initiation region, thus converting *BnSHP1.A9* from an active state to a silenced state. In this study, hypermethylation of the *BnSHP1.A9* promoter region, mainly through CG and CHH methylations, appears to be the major epigenetic factor in the regulation of gene expression. It seems reasonable for plants to evolve such an epigenetic regulatory mechanism to gain function for pod shatter resistance.

### Application of BnSHP1.A9 gene for oilseed rape breeding

In this study we investigated the molecular basis of pod shatter resistance in oilseed rape utilizing natural variation in *B. napus* germplasm and showed that a single gene, *BnSHP1.A9*, controls genetic variation for pod shatter resistance at the *qSRI.A9.1* locus. Our research provides two gene-specific markers, one co-dominant marker (IF4) detecting a sequence difference within the CDS of *BnSHP1.A9*, and the other co-dominant marker (BnSHP1.A9p1/p2) detecting presence/absence variation of LTR insertion in the *BnSHP1.A9* promoter region. Both markers can be applied for the efficient selection of this QTL for pod shatter resistance in oilseed rape breeding programs starting from early generations. Previously, there were only linked markers reported for marker-assisted selection of pod shatter resistance in oilseed rape (Raman *et al.*, 2014; Liu *et al.*, 2016). Our two gene-specific markers developed in this study could be used to further improve the resistance level by introducing the *BnSHP1.A9* (R2) allele with positive effect into lines containing the *BnSHP1.A9* (R1) allele for varietal improvement. These markers could be easily assayed via conventional agarose gel/high throughput capillary electrophoresis and KASP assays. The sources of pod shatter resistance identified herein can be used for introgression of favorable alleles and enrichment of alleles in the breeding germplasm.

Recently CRISPR–Cas9 genome editing and other genetic transformation platforms have become available for oilseed rape improvement (Zaman *et al.*, 2019). Editing *IND* and *ALC* genes has improved the pod shatter resistance in oilseed rape Braatz *et al.* (2018*a*, *b*). Our results clearly revealed that down-regulation of *BnSHP1.A9* could increase the PSRI. Genome editing can not only mutate *BnSHP1.A9*, but also other functionally redundant homologs of *SHP1*, as well as the homologs of *SHP2*. Knockdown of the multiple copies of functionally redundant genes or homologs has more effect on phenotypic variation. However, these approaches are difficult to deploy commercially due to the legal restrictions on genetically modified crops in some European countries. In countries that are open to gene-edited crops, our finding on the down-regulation of *BnSHP1.A9* having a positive effect on pod shatter resistance can be used to manipulate gene expression by generating mutation in this gene and its homologs for the improvement of pod shatter resistance in oilseed rape and reduce production losses.

In conclusion, we showed that a *SHP1* paralog controls pod shatter resistance at the *qSRI.A9.1* QTL in the DH population derived from R1/R2 and in a diversity panel of oilseed rape. Our findings suggest that a retrotransposon insertion contributes to pod shatter resistance by silencing the expression of *BnSHP1.A9* epigenetically via DNA methylation. Overall, this study provides a novel source of germplasm, gene-specific markers, and insights on the molecular basis of *SHP1*-mediated resistance to pod shatter in oilseed rape. These resources will facilitate the genetic improvement of pod shattering resistance in oilseed rape.

## Supplementary data

Supplementary data are available at *JXB* online.

Fig. S1. *BnSHP1.A9* expression analysis of leaves in the five T1 lines by RT-PCR.

Fig. S2. The phenotypes of the R1 and *BnSHP1.A9* overexpression transgenic plants.

Table S1. The *BnSHP1.A9* promoter and CDS (IF4) genotypes detected in the natural population.

Table S2. The primers used to amplify genomic sequences, CDS and spatial and temporal expression of *BnSHP1.A9*.

Table S3. The primer information of SSR and InDel markers used for genotyping a doubled haploid mapping population derived from the R1/R2 and saturation of marker density of A9 linkage group.

Table S4. McrBC digestion chop-PCR primers with gDNA template from R1, R2 20 DAF silique.

Table S5. The primer information for bisulfite sequencing regions spanning the upstream region of *BnSHP1.A9* promoter.

Table S6. The primer information for hygromycin resistance gene used to screen T0 and T1 transgene positive lines.

Table S7. The *BnSHP1.A9* SNP/Indel variations that were detected between R1 and R2 parental lines.

Table S8. The detailed information on the development of the IF4 marker used for genetic analysis of R1/R2 DH population.

Table S9. Pod shatter resistance index (PSRI) of transgenic and parental lines of R1/R2 doubled haploid population.

eraa281_suppl_Supplementary_Table_S1

eraa281_suppl_Supplementary_Table_S2

eraa281_suppl_Supplementary_Table_S3

eraa281_suppl_Supplementary_Table_S4

eraa281_suppl_Supplementary_Table_S5

eraa281_suppl_Supplementary_Table_S6

eraa281_suppl_Supplementary_Table_S7

eraa281_suppl_Supplementary_Table_S8

eraa281_suppl_Supplementary_Table_S9

eraa281_suppl_Supplementary_Figures

eraa281_suppl_Supplementary_Data

## Data Availability

Genome and cDNA sequences of BnSHP1.A9 were deposited in the NCBI database (ID: 2356618 and 2356745) and the information about markers is included in the supplementary files.
