# Supplementary material for: A copia-like retrotransposon insertion in the upstream region of the SHATTERPROOF1 gene, BnSHP1.A9, is associated with quantitative variation in pod shattering resistance in oilseed rape
Source: J Exp Bot. 2020 Jun 11;71(18):5402–13. doi: 10.1093/jxb/eraa281 (PMC7501816; doi:10.1093/jxb/eraa281)

Supplemental Fig. S1. *BnSHP1.A9* expression analysis of leaves in the 5 T1 lines by RT-PCR. *BnActin* gene was used as an internal control for relative expression analysis. 1, 2, 3: three individual plants of T18: 4, 5, 6: T24; 7, 8, 9: T26; 10, 11, 12: T33; 13, 14, 15: T9.

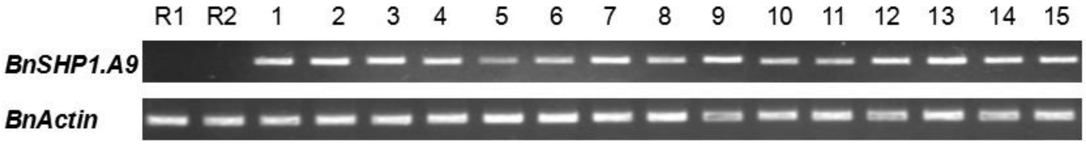

Supplemental Fig. 2. The phenotypes of the R1 and *BnSHP1.A9* overexpression transgenic plants.

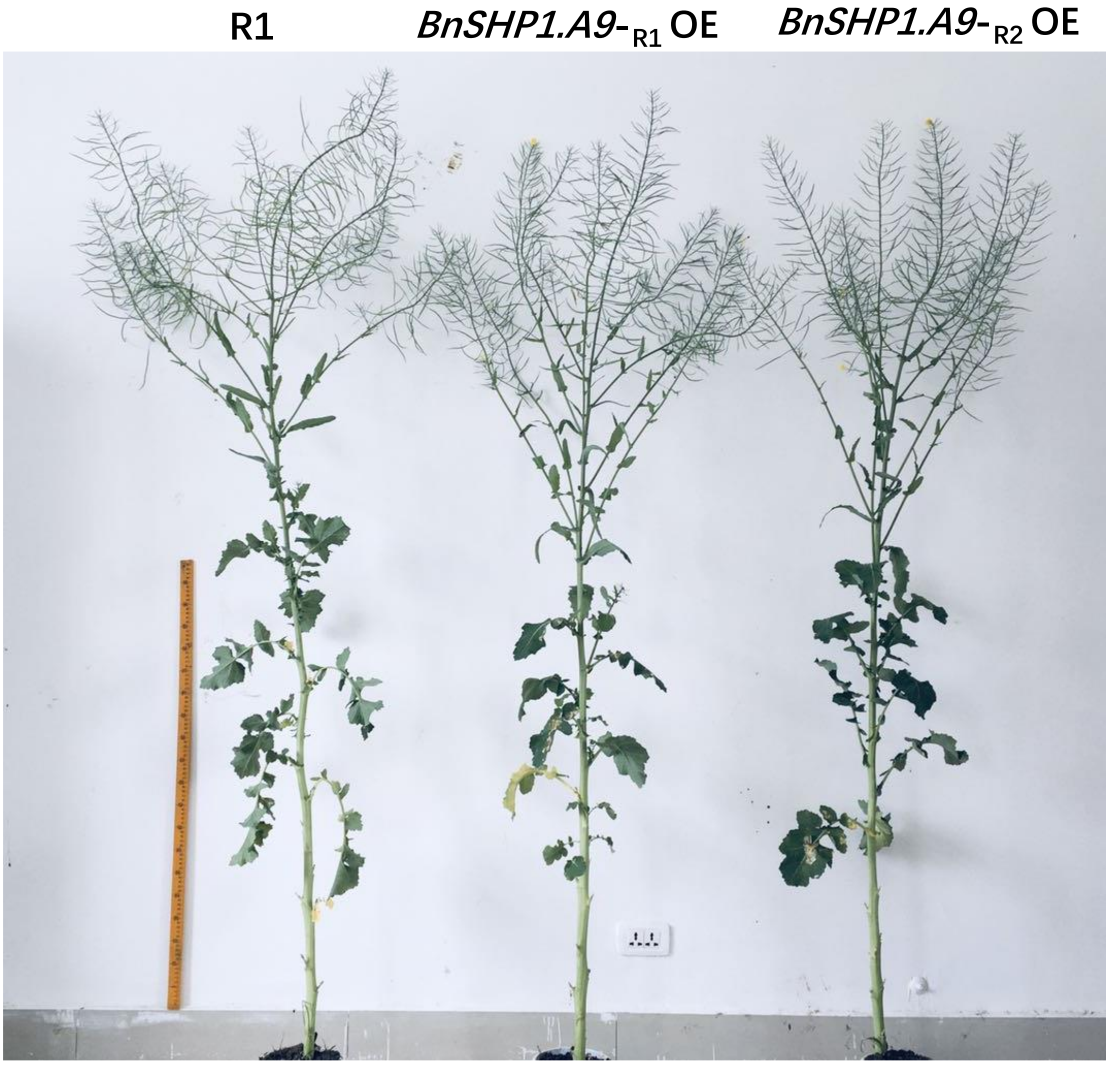

Supplement: eraa281_suppl_Supplementary_Figures [file eraa281_suppl_supplementary_figures.pdf]
