# Supplementary material for: A copia-like retrotransposon insertion in the upstream region of the SHATTERPROOF1 gene, BnSHP1.A9, is associated with quantitative variation in pod shattering resistance in oilseed rape
Source: J Exp Bot. 2020 Jun 11;71(18):5402–13. doi: 10.1093/jxb/eraa281 (PMC7501816; doi:10.1093/jxb/eraa281)
Supplement: eraa281_suppl_Supplementary_Data [file eraa281_suppl_supplementary_data.docx]

**Supplementary Data**

Supplementary data are available at *JXB* online.

Table S1. The *BnSHP1.A9* promoter and CDS (IF4) genotypes detected in the natural population.

Table S2. The primers used to amplify genomic sequences, CDS and spatial and temporal expression of *BnSHP1.A9*.

Table S3. The primers information of SSR and InDel markers used for genotyping a doubled haploid mapping population derived from the R1/R2 and saturation of marker density of A9 linkage group.

Table S4. McrBC digestion chop-PCR primers with gDNA template from R1, R2 20 DAF silique.

Table S5. The primers information of bisulfite sequencing regions spanning the upstream region of *BnSHP1.A9* promoter.

Table S6. The primers information of hygromycin resistance gene used to screen T0 and T1 transgene positive lines.

Table S7. The *BnSHP1.A9* SNP/Indel variations that were detected between R1 and R2 parental lines.

Table S8. The detailed information on the development of the *BnSHP1.A9* IF4 marker used for genetic analysis of R1/R2 DH population.

Table S9. Pod shatter resistance index (PSR1) of transgenic and parental lines of R1/R2 doubled haploid population. PSRI was tested using random impact test.

Fig. S1. *BnSHP1.A9* expression analysis of leaves in the 5 T1 lines by RT-PCR.

Fig. S2. The phenotypes of the R1 and *BnSHP1.A9* overexpression transgenic plants.
